# Juvenile morphology of seven intertidal mite species (Acari, Oribatida, Ameronothroidea) from the East Asian region

**DOI:** 10.1080/01647954.2021.1965656

**Published:** 2021-08-22

**Authors:** Tobias Pfingstl, Shimpei F. Hiruta, Wataru Hagino, Satoshi Shimano

**Affiliations:** a Institute of Biology, University of Graz, Graz, Austria; b Center for Molecular Biodiversity Research, National Museum of Nature and Science, Tsukuba, Japan; c Department of Bioresources Engineering, National Institute of Technology, Okinawa College, Nago City, Japan; d Science Research Center, Hosei University, Tokyo, Japan; e International Center for Island Studies Amami Station, Kagoshima University, Kagoshima, Japan; f Graduate School of Science, Tokyo Metropolitan University, Tokyo, Japan

**Keywords:** Nymphs, ontogeny, development, Japan, Fortuyniidae, Selenoribatidae

## Abstract

The present paper provides information on juvenile stages of seven intertidal oribatid mite species from the East Asian region. Larva and nymphs of the fortuyniid species *Fortuynia churaumi, F. shibai, F. rotunda* and *Alismobates reticulatus* are described and details about juvenile morphology of the selenoribatid species, *Arotrobates granulatus, Indopacifica taiyo* and *I. tyida* are given. Juveniles of the genus *Arotrobates* are reported for the first time and strong ventral femoral carinae are supposed to be diagnostic characters. Basically, the conservative morphology of immature stages could be confirmed in every studied taxon and its importance for phylogenetic considerations is emphasized.

## Introduction

Much of the life of oribatid mites is spent as a juvenile and therefore knowledge about these active developmental stages is important for reasons relating to ecology and systematics (Travé 1964). Grandjean (1954) argued that juvenile morphology of oribatid mites is more conservative than that of the adults and thus better suited to infer phylogenetic relationships. Zhang (2018) also emphasized that immature instars show a diversity of traits and other information that could be potentially useful for understanding mite taxonomy, phylogeny and biology. Despite these facts, juveniles of most species of mites are still unknown. For example, in oribatid mites (Astigmata excluded) juvenile instars were studied in only about 8% of all known species and as a consequence, immature morphology is completely unknown for many families (Norton and Ermilov 2014). Reasons for this tremendous lack of knowledge are that resources for making proper adult-juvenile associations are scarce (Norton and Ermilov 2014), and that rearing experiments in the laboratory require living specimens, considerable efforts, time and luck.

The intertidal oribatid mite families Fortuyniidae and Selenoribatidae represent two of the few exceptions, as juvenile morphology played an important role in early systematic considerations. Moreover, numerous species descriptions provided additional information on immatures resulting in a considerable amount of knowledge about juvenile morphology. Van der Hammen (1963) erected the Fortuyniidae based on juvenile morphology, and when Schuster (1963) proposed the Selenoribatidae, he also gave information on the juveniles of *Thalassozetes riparius* Schuster, 1963, which he described in this work. Shortly afterwards, Grandjean (1966, 1968) provided diagnoses for the latter family and these included juvenile characteristics, which he also used for a thorough comparison with the Fortuyniidae. Another important study using the juvenile morphology of these families for phylogenetic considerations, was that of Behan-Pelletier (1997), where she provided strong arguments supporting a relationship among Tegeocranellidae, Fortuyniidae and Selenoribatidae. This relationship was further corroborated by Pfingstl and Schuster (2012) using new data on fortuyniid juveniles. With the knowledge of these important studies, it was easier for experts to identify juveniles contained in a sample and to make the correct adult-juvenile association, which resulted in a relatively high number of described juveniles in Fortuyniidae and Selenoribatidae. Both families contain 63 species together and juvenile stages are known from 25 of these, which accounts for approx. 40%.

At the moment, 14 species of Fortuyniidae and Selenoribatidae have been described from Chinese and Japanese coastlines but immatures are known in none of them, except for *Circellobates venustus*, where Luxton (1992) only provided four sketchy figures of tritonymphal features that he described in two short sentences in his text. Apart from these insufficient data, most of the species were only found once in a single location with very few specimens (Luxton 1992; Karasawa and Aoki 2005), i.e. *Arotrobates lanceolatus* Luxton, 1992, *Psednobates uncunguis* Luxton, 1992, *Fortuynia sinensis* Luxton, 1992, *Circellobates venustus* Luxton, 1992 and *Alismobates rotundus* Luxton, 1992, *Rhizophobates shimojanai* Karasawa & Aoki, 2005, *Schusteria nagisa* Karasawa & Aoki, 2005 and *Schusteria saxea* Karasawa & Aoki, 2005, which could explain the lack of juveniles and thus the lack of knowledge. Other species were reported on several occasions and in different areas indicating high abundances and wider distributions for example, *Arotrobates granulatus* Luxton, 1992 and *Alismobates reticulatus* Luxton, 1992 which were both discovered in Hong Kong (Luxton 1992) and later found on several Japanese islands (Karasawa and Aoki 2005; Pfingstl et al. 2019b, 2021), or *Fortuynia rotunda* which was described from Mozambique (Marshall and Pugh 2002) and later reported from the Japanese Ryukyu islands (Karasawa and Aoki 2005), and *Fortuynia shibai* Aoki, 1974 which was originally found in a single location on the island of Shikoku (Aoki 1974) and later it was reported from various Japanese islands (Pfingstl et al. 2019b). In some of these cases, juveniles may have been present in the samples but were either not recognized or simply ignored and consequently they were discarded or stowed unnoticed in a collection. *Fortuynia churaumi* Pfingstl, Shimano & Hiruta, 2019, *Indpacifica taiyo* Pfingstl, Shimano & Hiruta, 2021 and *Indopacifica tyida* Pfingstl, Shimano & Hiruta, 2021, on the other hand, were described very recently (Pfingstl et al. 2019b, 2021), whereas these descriptions were accompanied by comprehensive morphometric and molecular genetic investigations that prevented the inclusion of detailed juvenile data in the first place.

Nevertheless, samples used for the latter two studies contained numerous juvenile specimens from these and several aforementioned species. Therefore, it is the aim of the present paper to provide detailed descriptions of the juvenile stages of these species, which are *F. churaumi, F. shibai, F. rotunda, Alismobates reticulatus, Arotrobates granulatus, I. taiyo* and *I. tyida*. Providing this important data is supposed to further contribute to the knowledge about ameronothroid juvenile morphology, which should represent a comprehensive basis for future phylogenetic considerations and reconstructions.

## Materials and methods

Samples of littoral algae were scraped off rocks, concrete walls and mangrove roots with a small shovel and then put in Berlese-Tullgren funnels for 12 to 24 hours to extract mites. Afterwards, specimens were preserved in ethanol (100%) for morphological investigation. Most samples were collected by the authors, and different collectors are given next to the respective sample. Juveniles were assigned to a specific species only if adults of a single species were contained in the respective sample.

### Sample locations

- *Fortuynia churaumi* – (1) Ishigaki-jima, pref. Okinawa: Yamabare, thick cushions of *Bostrychia* on large rocks; 24°26ʹ56.11”N 124°10ʹ46.41”E; 20 March 2019. (2) Okinawa-jima, pref. Okinawa: Sosu, *Bostrychia* in large crack on huge single rock; 26°48ʹ30.23”N 128°19ʹ6.00”E; 22 March 2019.
- *Fortuynia shibai –* (1) Omi-shima, pref. Ehime: black intertidal algae on mussels and barnacles on concrete wall; 34°13ʹ26.60”N 132°59ʹ0.68”E; 17 September 2018. (2) Kii-hantō, pref. Wakayama: Shirahama-cho, Nishinmuro-gun, Seto Marine Biological Laboratory, Field Science Education and Research Center; 33°41ʹ32”N 135°20ʹ04”E; 18 June 2019.
- *Fortuynia rotunda* – Iriomote-jima, pref. Okinawa: Estuary of Goyoshi river, brown algae on mangrove floor and roots, 24°19ʹ17”N 123°54ʹ38”E; 16 March 2019.
- *Alismobates reticulatus* – (1) Iriomote-jima, pref. Okinawa: Estuary of Goyoshi river, brown filamentous algae on mangrove floor, 24°19ʹ17.43”N 123°54ʹ38.87”E; 16 March 2019. Nadara river estuary, *Bostrychia* and other algae from boulder; 24°23ʹ52.93”N 123°49ʹ42.72”E; 16 March 2019. (2) Yonaguni-jima, pref. Okinawa: Kataburu Beach, *Bostrychia* and smaller plants on small rocks; 24°26ʹ25.78”N 122°58ʹ27.44”E; 18 March 2019. (3) Ishigaki-jima, pref. Okinawa: Kabira Bay, algae on rocks; 24°26ʹ56.2”N 124°08ʹ47.3”E; 31 March 2019; leg. H. Uchida.
- *Alismobates granulatus* – (1) Amami-oshima, pref. Kagoshima: Koniya Port, *Bostrychia* from small crevices on rough boulder; 28° 8ʹ51.19”N 129°18ʹ18.99”E; 24 March 2019. (2) Ishigaki-jima, pref. Okinawa: Kabira, unknown algae on rocks; 24°28ʹ25.7”N 124°08ʹ10.5”E; 31 March 2019; leg. H. Uchida.
- *Indopacifica taiyo* – (1) Yonaguni-jima, pref. Okinawa: Kataburu Beach, *Bostrychia* on cliffs; 24°26ʹ19.18”N 122°58ʹ21.81”E; 18 March 2019. (2) Amami-oshima, pref. Kagoshima: Isu Bay, *Bostrychia* on large boulder; 28°10ʹ12.79”N 129°21ʹ34.81”E; 24 March 2019.
- *Indopacifica tyida –* (1) Yonaguni-jima, pref. Okinawa: Sonai, dark black filamentous algae on rocks; 24°28ʹ15.69”N 122°59ʹ55.38”E; 19 March 2019.

### Preparation and visual documentation

Preserved specimens were embedded in Berlese mountant for microscopic analysis in transmitted light. Drawings were performed with an Olympus BH-2 Microscope equipped with a drawing attachment, then they were scanned and afterwards processed and digitized with the free and open-source vector graphics editor Inkscape (https://inkscape.org) and finally they were further modified with Adobe Photoshop CS.

Morphological terminology used in this paper follows that of Grandjean (1954, 1966, 1968) and Norton and Behan-Pelletier (2009).

## Results

### Description of juveniles

Family Fortuyniidae Hammen, 1963Genus *Fortuynia* Hammen, 1960

### Common features of juvenile stages

Apheredermous. Colour dark brown. Integument plicate and soft, except for centrodorsal plate. Prodorsum triangular, rostrum rounded, cerotegument overall finely granular. Rostral seta (*ro*) thin, long, lamellar seta (*le*) also thin but slightly shorter. Minute or alveolar exobothridial (*ex*) and interlamellar seta (*in*). Bothridium small cup-like and laterally opened. Sensillum short, clavate. Gnathosoma no obvious differences from adult stage. Gastronotum oval in dorsal view, slightly convex in lateral view. Posterior half of centrodorsal plate with lesser sclerotized area resembling inverted Y, present in nymphs. Large folds framing centrodorsal plate, showing fine granular surface. Orifice of opisthonotal gland *gla* located in posterior third of lateral folds. Legs with paraxial porous areas present on all femora.

***Fortuynia shibai*** Aoki, 1974

#### Larva

(N = 4): length 200–234 µm (mean 224 µm).

**Figure 1.**
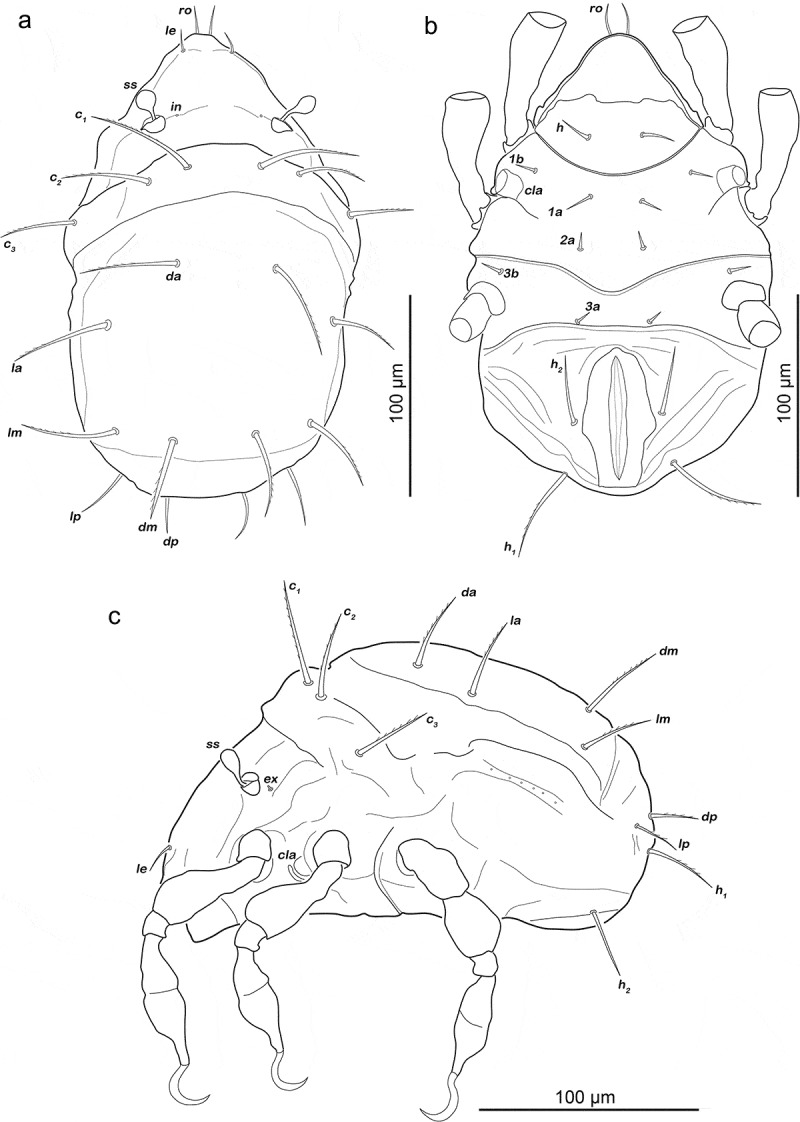
*Fortuynia shibai* larva. (a) dorsal view. (b) ventral view (distal legs segments and gnathosoma omitted). (c) lateral view (legs drawn without setation)

Prodorsum (Figure 1(a)). Interlamellar seta only alveolar, exobothridial seta minute.

Gastronotic region (Figure 1(a,c)). Eleven pairs of long notogastral setae (length 31–35 µm), *c_1-3_, da, dm, dp, la, lm, lp, h_1-2_*, all distally slightly serrate, except for *h2*. Transversal ridge on centrodorsal plate passing posterior line of setae *lm* and *dm*.

Podosoma and venter (Figure 1(b)). Epimeral setation 2-1-2, all setae short (approx. 8 µm) and spiniform. Claparède’s organ globular, nearly completely covered by epimeral cuticle. Aggenital, genital, adanal and anal setae not developed.

**Figure 2.**
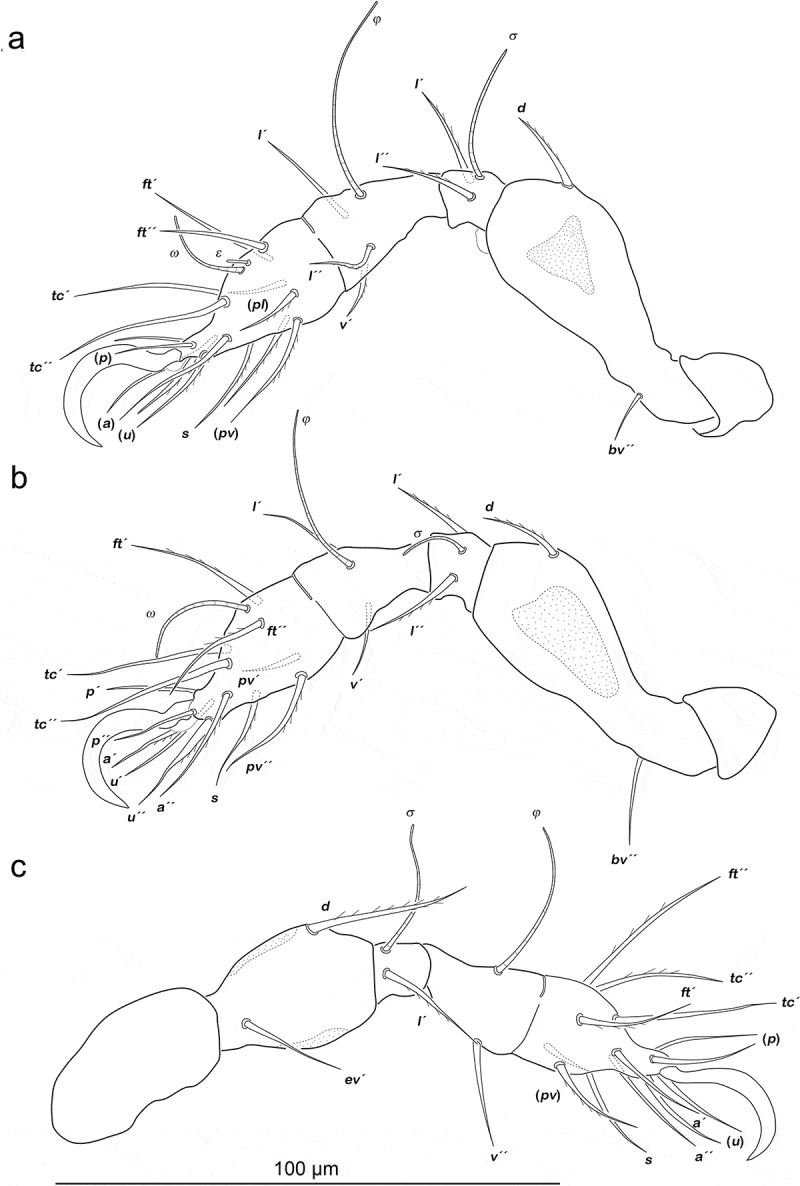
*Fortuynia shibai* larva, left legs antiaxial view. (a) Leg I. (b) Leg II. (c) Leg III. Scale bar valid for all legs

Legs (Figure 2). Femora I and II with large triangular porous areas on paraxial side. Two slender paraxial longitudinal porous areas on femur III, one dorsally the other ventrally. Setation and solenidia see Table 1.

**Table 1.** *Fortuynia shibai*, chaetome and solenidia from larva to adult. First development of setae characterized by letters. () = pairs of setae, – = no change with regard to preceding stage. *information taken from Pfingstl et al. (2019b)

	Instars	Trochanter	Femur	Genu	Tibia	Tarsus	Chaetome	Solenidia
Leg I	larva	-	*d, bv´´*	(*l), σ*	(*l), v´, φ_1_*	(*pl*), (*pv), s*, (*a*), (*u*), (*p*), (*tc*), (*ft), ε, ω_1_*	0-2-2-3-16	1-1-1
	protonymph	-	-	-	-	*ω_2_*	0-2-2-3-16	1-1-2
	deutonymph	-	*l´*	-	*φ_2_*	-	0-3-2-3-16	1-2-2
	tritonymph	-	*l´´*	-	-	(*it*)	0-4-2-3-18	1-2-2
	adult*	*l´´*	-	-	-	-	1-4-2-3-18	1-2-2
Leg II	larva	-	*d, bv´´*	(*l*), *σ*	*l´, v´,φ*	(*pv), s*, (*a*), (*u*), (*p*), (*tc*), (*ft), ω*	0-2-2-2-13	1-1-1
	protonymph	-	-	-	-	-	0-2-2-2-13	1-1-1
	deutonymph	-	*l´´*	-	-	-	0-3-2-2-13	1-1-1
	tritonymph	-	*l´*	-	-	(*it*)	0-4-2-2-15	1-1-1
	adult*	*l´´*	-	-	*l´´*	-	1-4-2-3-15	1-1-1
Leg III	larva	-	*d*, e*v´*	*l´, σ*	*v´, φ*	(*pv), s*, (*a*), (*u*), (*p*), (*tc*), (*ft*)	0-2-1-1-13	1-1-0
	protonymph	-	-	-	-	-	0-2-1-1-13	1-1-0
	deutonymph	*v´*	-	-	-	-	1-2-1-1-13	1-1-0
	tritonymph	-	*l´*	-	*l´*	(*it*)	1-3-1-2-15	1-1-0
	adult*	*l´*	-	-	*l´´*	-	2-3-1-3-15	1-1-0
Leg IV	protonymph	-	-	-	-	(*pv*), (*u*), (*p), ft´*	0-0-0-0-7	0-0-0
	deutonymph	-	*d*, e*v´*	*d, l´*	*v´, φ*	*s*, (*a*), (*tc*)	0-2-2-1-12	0-1-0
	tritonymph	*v´*	-	-	*l´*	-	1-2-2-2-12	0-1-0
	adult*	-	-	-	*l´´*	-	1-2-2-3-12	0-1-0

#### Protonymph

(N = 10): length 277–313 (mean 292 µm).

**Figure 3.**
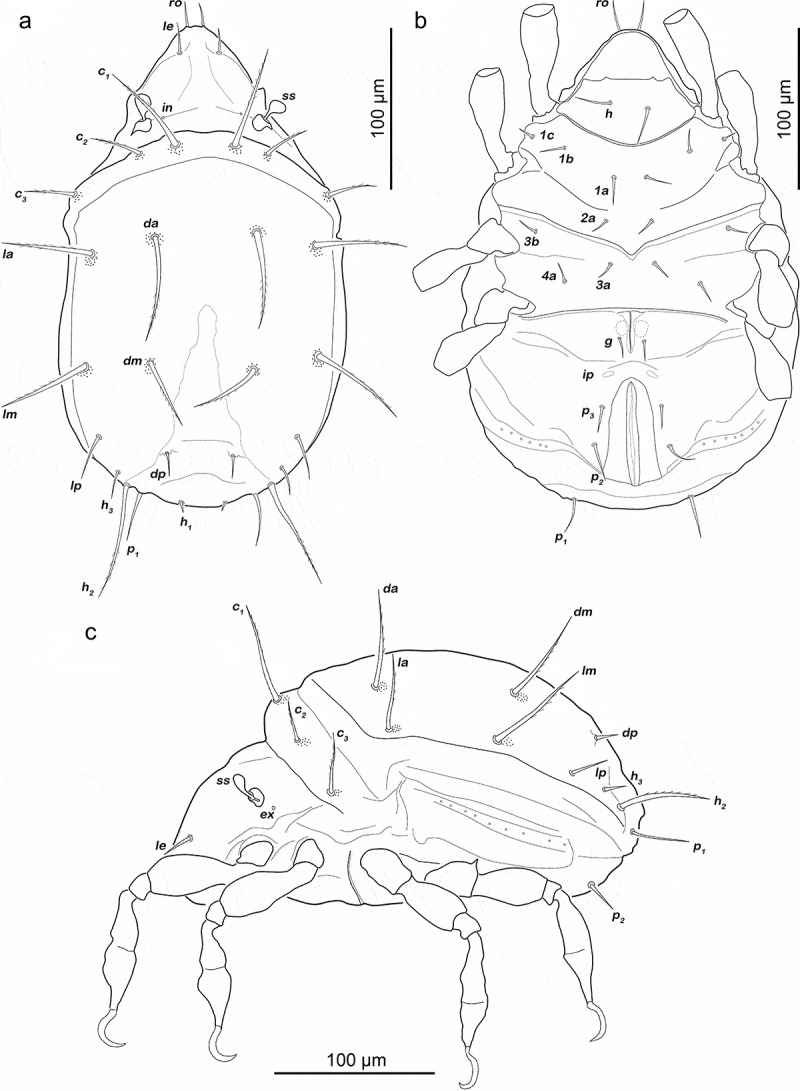
*Fortuynia shibai* protonymph. (a) dorsal view. (b) ventral view (distal legs segments and gnathosoma omitted). (c) lateral view (seta *h_1_* covered by seta *h_2_* and therefore not indicated; legs drawn without setation)

Gastronotic region (Figure 3(a,c)). Fifteen pairs of notogastral setae; *c_1-3_, da, dm, dp, la, lm, lp, h_1-3_, p_1-3_*. Seta *c_1_, da, dm, la, lm, h_2_* long (43–62 µm) and distally slightly barbed, *c_2_, c_3_, lp* spiniform normal (22–31 µm), *dp, h_3_* short (ca. 8 µm) and *h_1_* very short (ca. 4 µm). Setae *p_1-3_* spiniform, normal length (20–30 µm).

Podosoma and venter (Figure 3(b)). Epimeral setation 3-1-2-1, all setae spiniform and approx. same length (6–9 µm). Seta *1 c* and *4a* added in this stage. One pair of short genital setae *g_1_*. Adanal and anal setae not developed. Cupule of lyrifissure *ip* anterior of anal opening.

**Figure 4.**
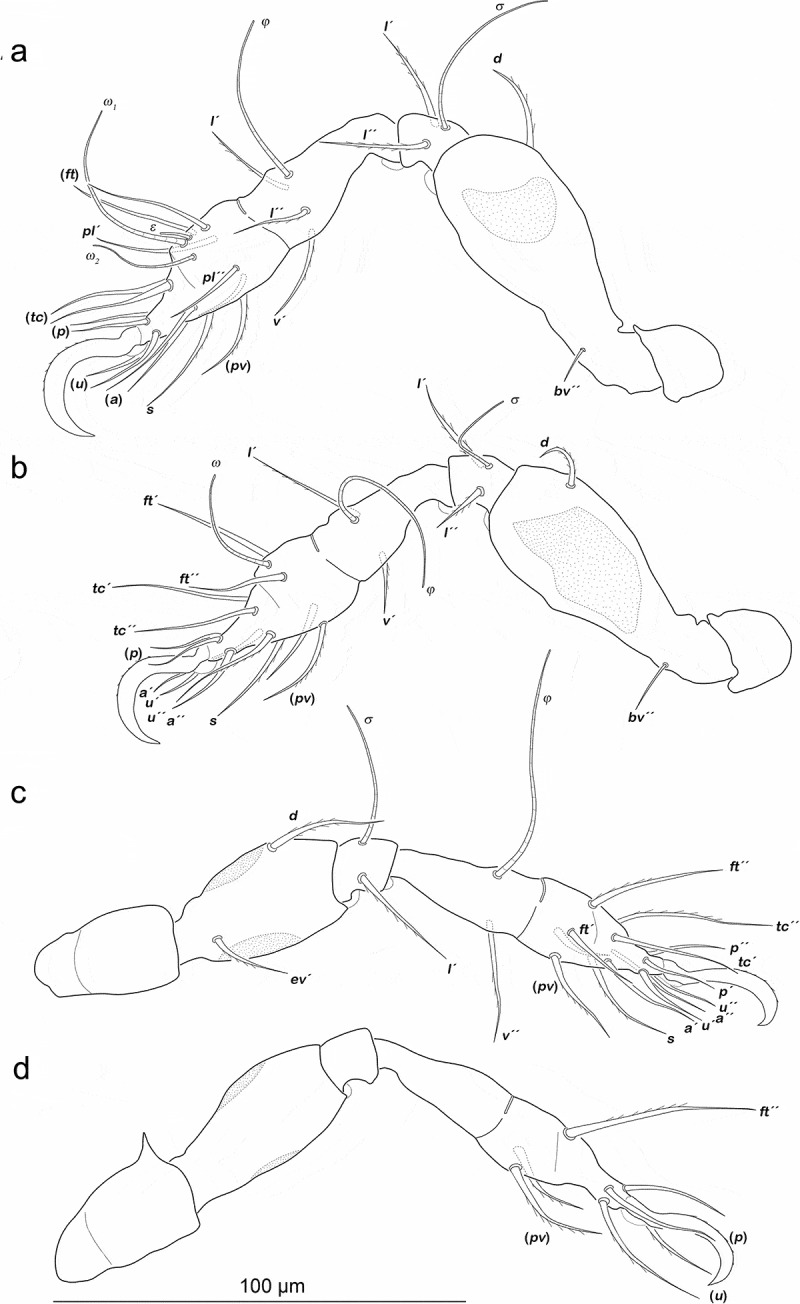
*Fortuynia shibai* protonymph, left legs antiaxial view. (a) Leg I. (b) Leg II. (c) Leg III. (d) Leg IV. Scale bar valid for all legs

Legs (Figure 4). Porous areas on legs I–III same as in larva. Femur IV with two paraxial longitudinal porous areas, one dorsally, one ventrally. Setation and solenidia are shown in Table 1.

#### Deutonymph

(N = 14): length 344–406 µm (mean 364 µm).

**Figure 5.**
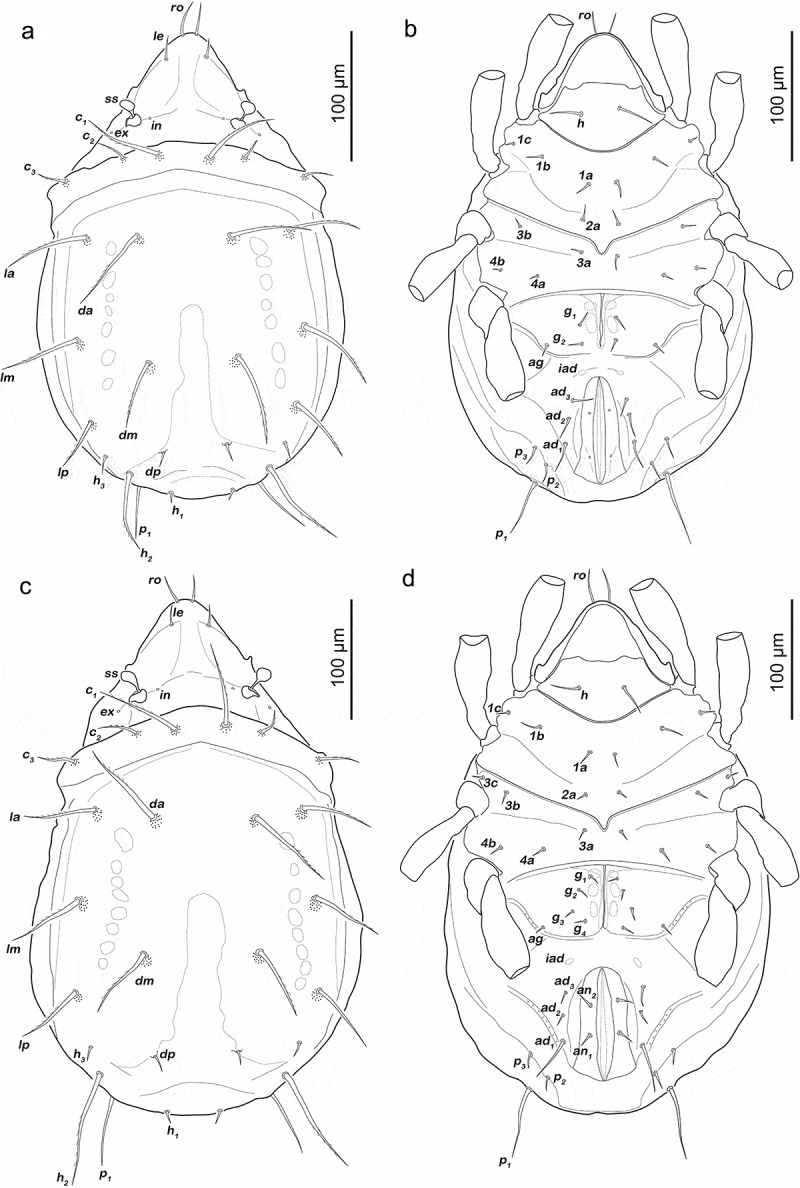
*Fortuynia shibai* nymphs. (a) deutonymph dorsal view. (b) deutonymph ventral view (distal leg segments and gnathosoma omitted). (c) tritonymph dorsal view. (d) tritonymph ventral view (distal leg segments and gnathosoma omitted)

Gastronotic region (Figure 5(a)). Fifteen pairs of notogastral setae, same positions and shapes as in protonymph, except for *p_1-3_* positioned more laterally, *p_1_* longest (102–114 µm), *p_2-3_* short (11–13 µm).

Podosoma and venter (Figure 5(b)). Epimeral setation 3-1-2-2, seta *4b* added in this stage. Two pairs of short genital setae *g_1-2_*. One pair of aggenital setae. Three pairs of adanal setae flanking anal orifice. Two pairs of vestigial anal setae. Cupule of lyrifissure *iad* anterior of anal opening.

**Figure 6.**
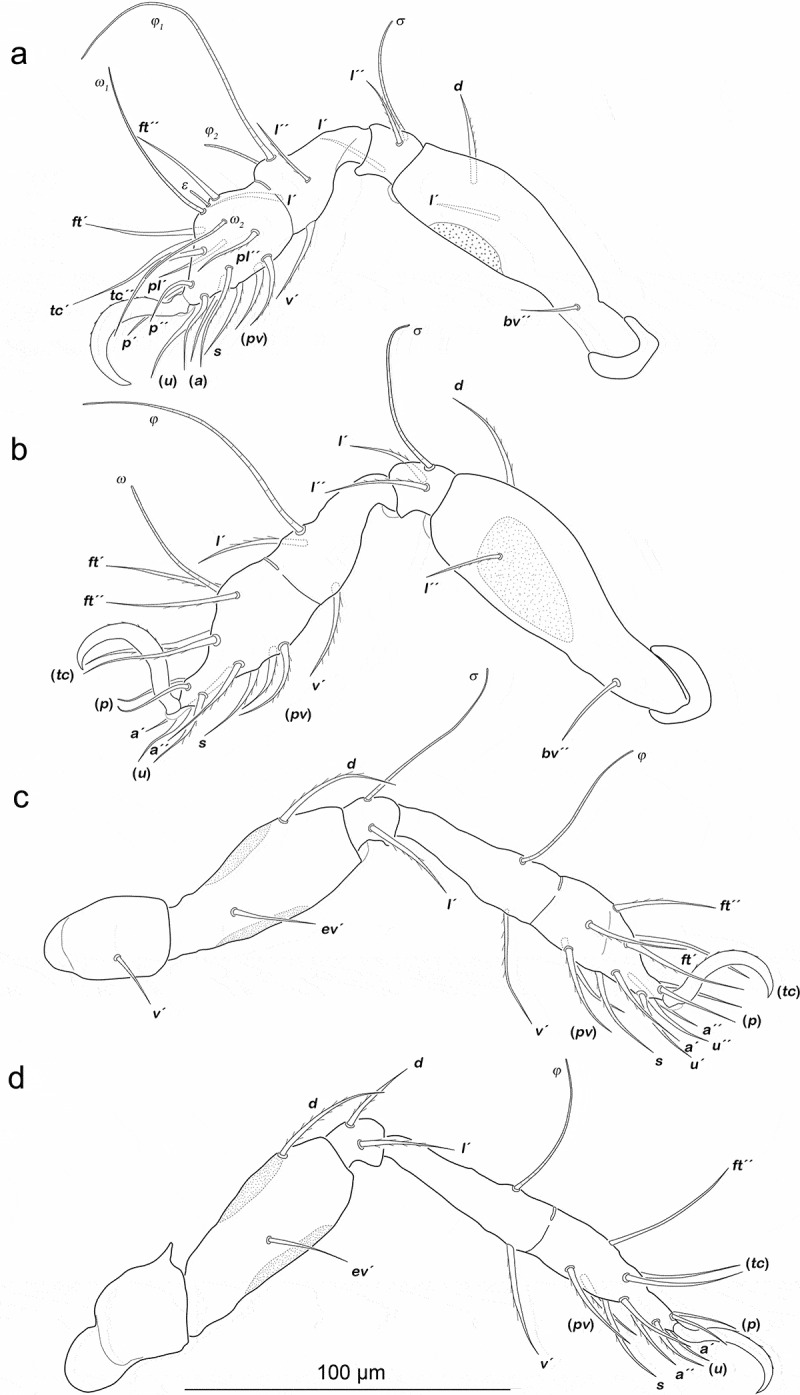
*Fortuynia shibai* deutonymph, left legs antiaxial view. (a) Leg I, femur slightly ventrally distorted. (b) Leg II. (c) Leg III. (d) Leg IV. Scale bar valid for all legs

Legs (Figure 6). Porous areas same as in protonymph. Setation and solenidia see Table 1.

#### Tritonymph

(N = 4): length 400–463 µm (mean 429 µm).

Gastronotic region (Figure 5(c)). Fifteen pairs of notogastral setae (12–115 µm), no difference to deutonymph.

Podosoma and venter (Figure 5(d)). Epimeral setation 3-1-3-2, seta *3 c* added in this stage, close to trochanter III. Four pairs of genital setae *g_1-4_*. Three pairs of adanal setae; *ad_2-3_* short (ca. 9 µm), *ad_1_* at least twice as long (ca. 40 µm). Two pairs of short anal setae (9–11 µm), fully developed in this stage.

Legs. Setation and solenidia are shown in Table 1.

### Remarks

The barbulation of notogastral setae shown in the adults of *F. shibai* is already present in the larval stage and remains constant throughout the development. *Fortuynia shibai* adults also show porous areas on all femora and trochanters III and IV. The femoral porous areas are already present in all juvenile stages but porous areas on trochanters could not be confirmed in any ontogenetic stage (either they are difficult to trace or they are only developed in the adult stage).

***Fortuynia churaumi*** Pfingstl, Shimano & Hiruta, 2019

Larva and protonymph unknown.

#### Deutonymph

(N = 1): length 350 µm.

Prodorsum. Interlamellar seta and exobothridial setae minute.

Gastronotic region. Fifteen pairs of normal spiniform notogastral setae; *c_1-3_, da, dm, dp, la, lm, lp, h_1-3_, p_1-3_*. All setae approx. same length (19–22 µm), except for *h_2_* considerably longer (31 µm).

**Figure 7.**
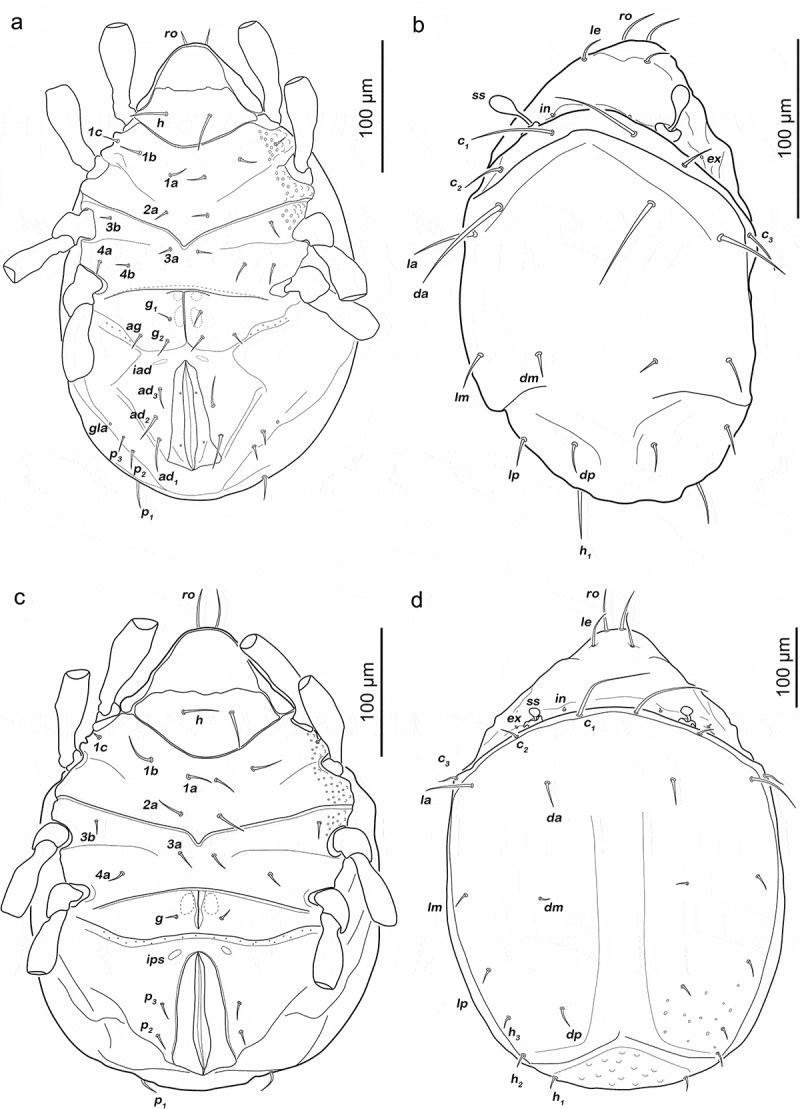
Japanese *Fortuynia* juveniles. (a) *F. churaumi*, deutonymph ventral view. (b) *F. rotunda*, larva dorsal view (slightly laterally rotated). (c) *F. rotunda*, protonymph ventral view (distal leg segments and gnathosoma omitted). (d) *F. rotunda*, tritonymph dorsal view

Podosoma and venter (Figure 7(a)). Epimeral setation 3-1-2-2, seta *4b* added in this stage. Two pairs of short genital setae *g_1-2_* (13–16 µm). One pair of aggenital setae. Three pairs of adanal setae flanking anal orifice. Two pairs of vestigial anal setae.

Legs. Setation and solenidia are shown in Table 2.

**Table 2.** *Fortuynia churaumi*, chaetome and solenidia from deutonymph to adult. First development of setae characterized by letters. () = pairs of setae, – = no change with regard to preceding stage. *information taken from Pfingstl et al. (2019a)

	Instars	Trochanter	Femur	Genu	Tibia	Tarsus	Chaetome	Solenidia
Leg I	deutonymph	-	*bv´´, l´, d*	(*l), σ*	*v´*, (*l), φ_1-2_*	(*pl*), (*pv), s*, (*a*), (*u*), (*p*), (*tc*), (*ft), ω_1-2_, ε*	0-3-2-3-16	1-2-2
	tritonymph	-	*l´´*	-	-	(*it*)	0-4-2-3-18	1-2-2
	adult*	*l´´*	-	-	-	-	1-4-2-3-18	1-2-2
Leg II	deutonymph	-	*bv´´, l´´, d*	(*l*), *σ*	*v´, l´, φ*	(*pv), s*, (*a*), (*u*), (*p*), (*tc*), (*ft), ω*	0-3-2-2-13	1-1-1
	tritonymph	-	*l´*	-	*l´´*	(*it*)	0-4-2-3-15	1-1-1
	adult*	*l´´*	-	-	-	-	1-4-2-3-15	1-1-1
Leg III	deutonymph	*v´*	*ev´, d*	*l´, σ*	*v´, φ*	(*pv), s*, (*a*), (*u*), (*p*), (*tc*), (*ft*)	1-2-1-1-13	1-1-0
	tritonymph	-	*l´*	-	*l´*	(*it*)	1-3-1-2-15	1-1-0
	adult*	*l´*	-	-	*l´´*	-	2-3-1-3-15	1-1-0
Leg IV	deutonymph	-	*d, ev´*	*d, l´*	*v´, φ*	(*pv*), *s*, (*a*), (*u*), (*p*), (*tc), ft*´´	0-2-2-1-12	0-1-0
	tritonymph	*v´*	-	-	*l´*	-	1-2-2-2-12	0-1-0
	adult*	-	-	-	*l´´*	-	1-2-2-3-12	0-1-0

#### Tritonymph

(N = 16): length 388–419 (mean 396 µm).

**Figure 8.**
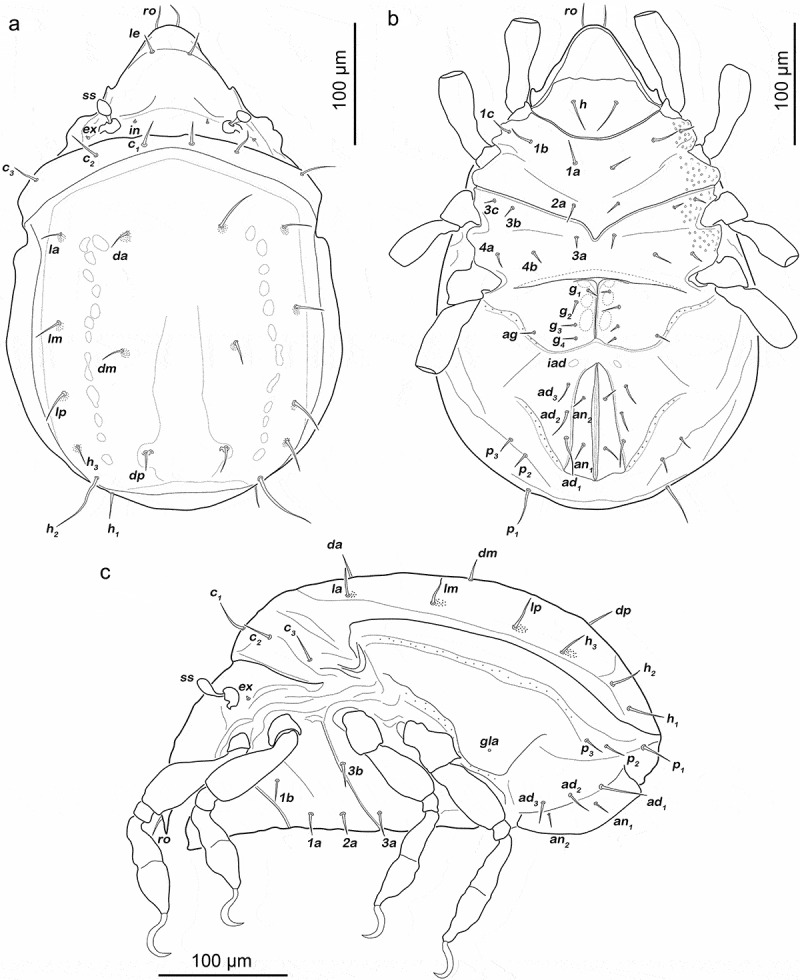
*Fortuynia churaumi* tritonymph. (a) Dorsal view. (b) ventral view (distal leg segments and gnathosoma omitted). (c) lateral view (legs drawn without setation)

Gastronotic region (Figure 8(a,c)). Fifteen pairs of notogastral setae (25–62 µm), no difference from deutonymph.

Podosoma and venter (Figure 8(b)). Epimeral setation 3-1-3-2, seta *3 c* close to trochanter III. Four pairs of genital setae *g_1-4_*. One pair of aggenital setae. Three pairs of adanal setae, *ad_1_* longest (ca. 28 µm). Two pairs of anal setae fully developed.

**Figure 9.**
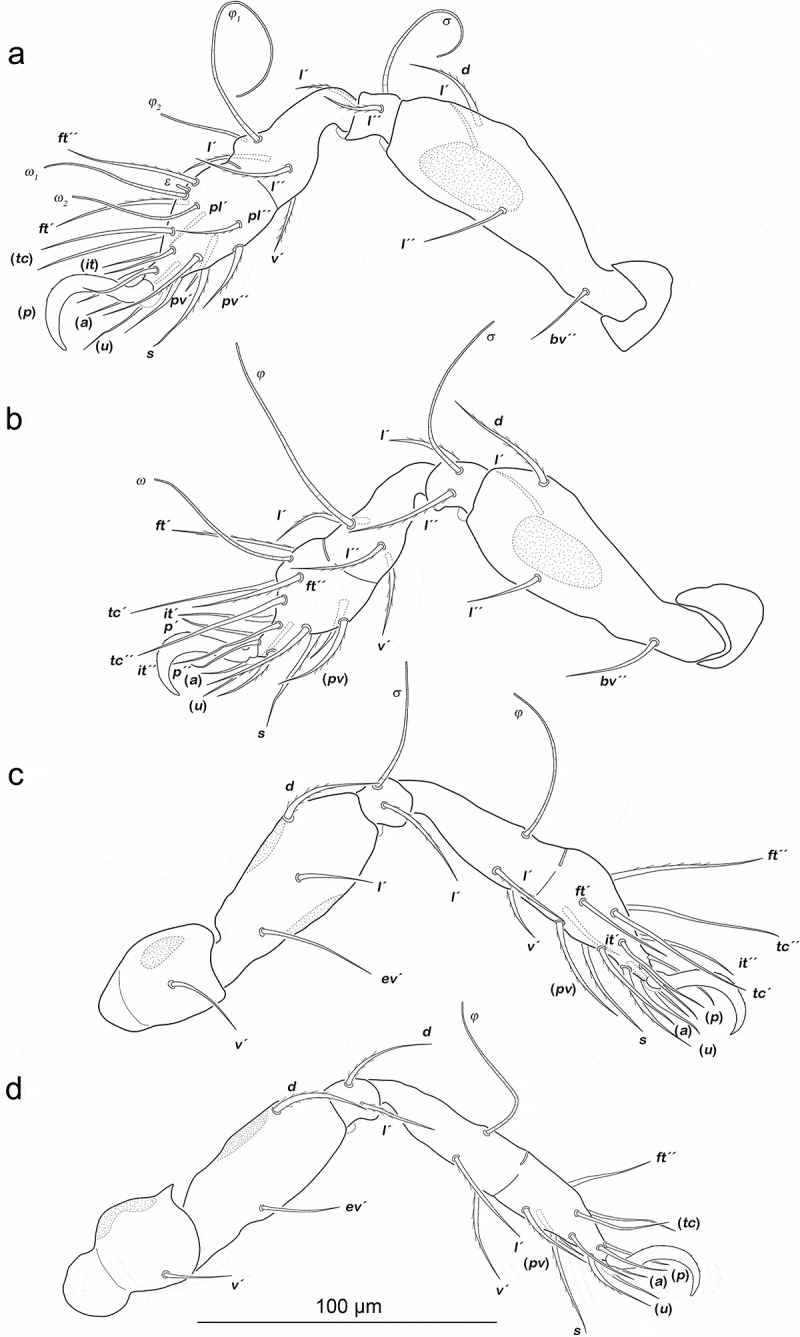
*Fortuynia churaumi* tritonymph, left legs antiaxial view. (a) Leg I. (b) Leg II. (c) Leg III. (d) Leg IV

Legs (Figure 9). Femora I and II with large elliptic porous areas on paraxial side. Two slender paraxial longitudinal porous areas on femur III, one dorsally the other ventrally. Elliptic or kidney-shaped porous areas on paraxial dorsal side of trochanter III–IV. Setation and solenidia shown in Table 2.

### Remarks

*Fortuynia churaumi* adults show a specific reticulate pattern (fine dense granulation interrupted by non-granular lines) on their femora (see Pfingstl et al. 2019b, Figure 3), this pattern is already observable in the tritonymph.

*Fortuynia shibai* and *F. churaumi* may occur syntopically in some locations on the Japanese islands (Pfingstl et al. 2019b). Adults can be easily distinguished and the same applies to juveniles allowing to easily separate them if contained in a single sample. *Fortuynia shibai* immatures always show distally barbed notogastral setae (vs. smooth in *F. churaumi*) and these setae are considerably longer than in *F. churaumi*.

***Fortuynia rotunda*** Marshall & Pugh, 2002

#### Larva

(N = 1): length 262 µm.

Prodorsum (7B). Interlamellar and exobothridial seta minute.

Gastronotic region (Figure 7(b)). Eleven pairs of smooth spiniform notogastral setae; *c_1-3_, da, dm, dp, la, lm, lp, h_1-2_*. Anterior notogastral setae (50–55 µm) three times longer than posterior notogastral setae (12–15 µm). Transversal ridge on centrodorsal plate passing posterior line of setae *lm* and *dm* medially incomplete.

Podosoma and venter. Epimeral setation 2-1-2. Claparède’s organ globular, nearly completely covered by epimeral cuticle. Aggenital, genital, adanal and anal setae not developed.

Legs. Setation and solenidia see Table 3.

**Table 3.** *Fortuynia rotunda*, chaetome and solenidia from larva to adult (except for deutonymph which is unknown)

	Instars	Trochanter	Femur	Genu	Tibia	Tarsus	Chaetome	Solenidia
Leg I	larva	-	*d, bv´´*	(*l), σ*	(*l), v´, φ_1_*	(*pl*), (*pv), s*, (*a*), (*u*), (*p*), (*tc*), (*ft), ε, ω_1_*	0-2-2-3-16	1-1-1
	protonymph	-	*l´*	-	-	*ω_2_*	0-3-2-3-16	1-1-2
	tritonymph	-	*l´´*	-	*φ_2_*	(*it*)	0-4-2-3-18	1-2-2
	adult	*v´*	-	-	-	-	1-4-2-3-18	1-2-2
Leg II	larva	-	*d, bv´´*	(*l*), *σ*	*l´, v´, φ*	(*pv), s*, (*a*), (*u*), (*p*), (*tc*), (*ft), ω*	0-2-2-2-13	1-1-1
	protonymph	-	*l´*	-	-	-	0-3-2-2-13	1-1-1
	tritonymph	-	*l´´*	-	-	(*it*)	0-4-2-2-15	1-1-1
	adult	*v´*	-	-	*l´´*	-	1-4-2-3-15	1-1-1
Leg III	larva	-	*d*, e*v´*	*l´, σ*	*v´, φ*	(*pv), s*, (*a*), (*u*), (*p*), (*tc*), (*ft*)	0-2-1-1-13	1-1-0
	protonymph	-	-	-	-	-	0-2-1-1-13	1-1-0
	tritonymph	*v´*	*l´*	-	*l´´*	(*it*)	1-3-1-2-15	1-1-0
	adult	*l´*	-	-	*l´*	-	2-3-1-3-15	1-1-0
Leg IV	protonymph	-	-	-	-	(*pv*), (*u*), (*p), ft´*	0-0-0-0-7	0-0-0
	tritonymph	*v´*	*d*, e*v´*	*d, l´*	*l´, v´, φ*	*s*, (*a*), (*tc*)	1-2-2-2-12	0-1-0
	adult	-	-	-	*l´´*	-	1-2-2-3-12	0-1-0

#### Protonymph

(N = 1): length 350 µm.

Gastronotic region. Fifteen pairs of simple notogastral setae; *c_1-3_, da, dm, dp, la, lm, lp, h_1-3_, p_1-3_*. Anterior notogastral setae (65–70 µm) approx. twice as long as posterior notogastral setae (25–30 µm).

Podosoma and venter (Figure 7(c)). Epimeral setation 3-1-2-1. One pair of short genital setae (11 µm). Adanal and anal setae not developed.

Legs. Setation and solenidia are shown in Table 3.

### Deutonymph unknown

#### Tritonymph

(N = 1): length 544 µm.

Gastronotic region (Figure 7(d)). Fifteen pairs of notogastral setae (30–100 µm), no conspicuous difference to protonymph.

Podosoma and venter. Epimeral setation 3-1-3-2, seta *3 c* added in this stage, close to trochanter III. Four pairs of genital setae. Three pairs of adanal setae. Two pairs of anal setae, fully developed in this stage.

Legs. Large porous areas on paraxial side of each femur. Porous areas on trochanters not traceable. Setation and solenidia see Table 3.

### Remarks

When Marshall and Pugh (2002) described *Fortuynia rotunda* from African coasts, they gave short information on the legs, e.g. colour and length of segments, and only details about the setae of genu I and II (p. 179, Figure 4(b)). Karasawa and Aoki (2005) provided a redescription of *F. rotunda* based on their specimens found on the Japanese Ryukyu islands but also gave very few information about the legs, e.g. lengths of segments, smoothness of claws. Accordingly, we present herein the setation and solenidia for the legs of this species for the first time in detail and although these formulas may be the same for all Japanese *F. rotunda*, they may differ in the African *F. rotunda*. Therefore, the development of leg setation given in the present paper is so far only valid for Japanese *F. rotunda*.

*Fortuynia rotunda* is characterized by its very rounded notogaster and the juveniles already show a similar body shape with the gastronotic region being considerably broad and rounded. However, this trait should not be considered diagnostic, neither for adults nor for juveniles because it is also present in all developmental stages of *F. smiti* (Pfingstl 2015). It is further assumable that other species with similarly rounded body shapes of adults, like *Fortuynia maculata*, show similar rounded larva and nymphs.

Genus *Alismobates* Luxton, 1992

### Common features of juvenile stages

Apheredermous. Colour dark brown. Integument plicate and soft, except for centrodorsal plate. Prodorsum triangular, anterior part finely granular, rostrum rounded. Rostral and lamellar seta short and simple. Minute exobothridial and interlamellar seta*e*. Sensillum clavate and distally spinose. Bothridium cup-like, laterally opened. Gnathosoma no obvious differences to adult stage. Hysterosoma slightly convex. Hysterosomal cupules not traceable in any stage. Large, stronger sclerotized centrodorsal plate bearing all notogastral setae, except for *c_1-3_* in all stages and seta *h_2_* in the larval stage. Centrodorsal plate showing either foveate or reticulate cuticular pattern. In nymphal stages posterior part of this plate showing a median lesser sclerotized and slightly concave conspicuous area without distinct surface pattern resembling an inverted Y. Large folds framing centrodorsal plate completely, showing fine granular surface. Orifice of opisthonotal gland (*gla*) located in posterior third of lateral folds. Ventral sejugal suture developed as distinct furrow, posterior border medially opened over a short distance. Legs monodactylous with large hook-like claws. Large porous areas on same leg segment as in adults.

***Alismobates reticulatus*** Luxton, 1992

Larva and protonymph unknown.

#### Deutonymph

(N = 1): length 222 µm.

Gastronotic region. Large centrodorsal plate showing reticulate surface pattern, except for inverted Y-shaped area on posterior third of gastronotic region. Fifteen pairs of short spiniform notogastral setae (8–10 µm); *c_1-3_, da, dm, dp, la, lm, lp, h_1-3_, p_1-3_*.

Podosoma and venter. Epimeral setation 3-1-2-1. Two pairs of short genital setae (5–6 µm). One pair of aggenital setae. Three pairs of adanal setae *ad_1-3_* flanking anal orifice. Two pairs of vestigial anal setae.

Legs. Setation and solenidia are shown in Table 4.

**Table 4.** *Alismobates reticulatus*, chaetome and solenidia from deutonymph to adult

	Instars	Trochanter	Femur	Genu	Tibia	Tarsus	Chaetome	Solenidia
Leg I	deutonymph	-	*d, bv´´*, (*l*)	(*l), σ*	(*l), v´, φ_1_, φ_2_*	(*pl*), (*pv), s*, (*a*), (*u*), (*p*), (*tc*), (*ft), ε, ω_1_, ω_2_*	0-4-2-3-16	1-2-2
	tritonymph	-	-	-	-	(*it*)	0-4-2-3-18	1-2-2
	adult	-	-	-	-	-	0-4-2-3-18	1-2-2
Leg II	deutonymph	-	*d, bv´´*, (*l*)	(*l*), *σ*	*l´, v´,φ*	(*pv), s*, (*a*), (*u*), (*p*), (*tc*), (*ft), ω*	0-4-2-2-13	1-1-1
	tritonymph	-	-	-	-	(*it*)	0-4-2-2-15	1-1-1
	adult	-	-	-	*l´´*	-	0-4-2-3-15	1-1-1
Leg III	deutonymph	-	*d, ev´*	*l´, σ*	*v´, φ*	(*pv), s*, (*a*), (*u*), (*p*), (*tc*), (*ft*)	0-2-1-1-13	1-1-0
	tritonymph	*v´*	-	-	-	(*it*)	1-2-1-1-15	1-1-0
	adult	-	*l´*	-	(*l*)	-	1-3-1-3-15	1-1-0
Leg IV	deutonymph	-	*d, ev´*	*d, l´*	*v´, φ*	(*pv*), (*u*), (*p*), s, (a), (tc), *ft´´*	0-2-2-1-12	0-1-0
	tritonymph	*v´*	-	-	*l´´*	-	1-2-2-2-12	0-1-0
	adult	-	-	-	*l´*	-	1-2-2-3-12	0-1-0

#### Tritonymph

(N = 4): length 262–283 (mean 276 µm).

**Figure 10.**
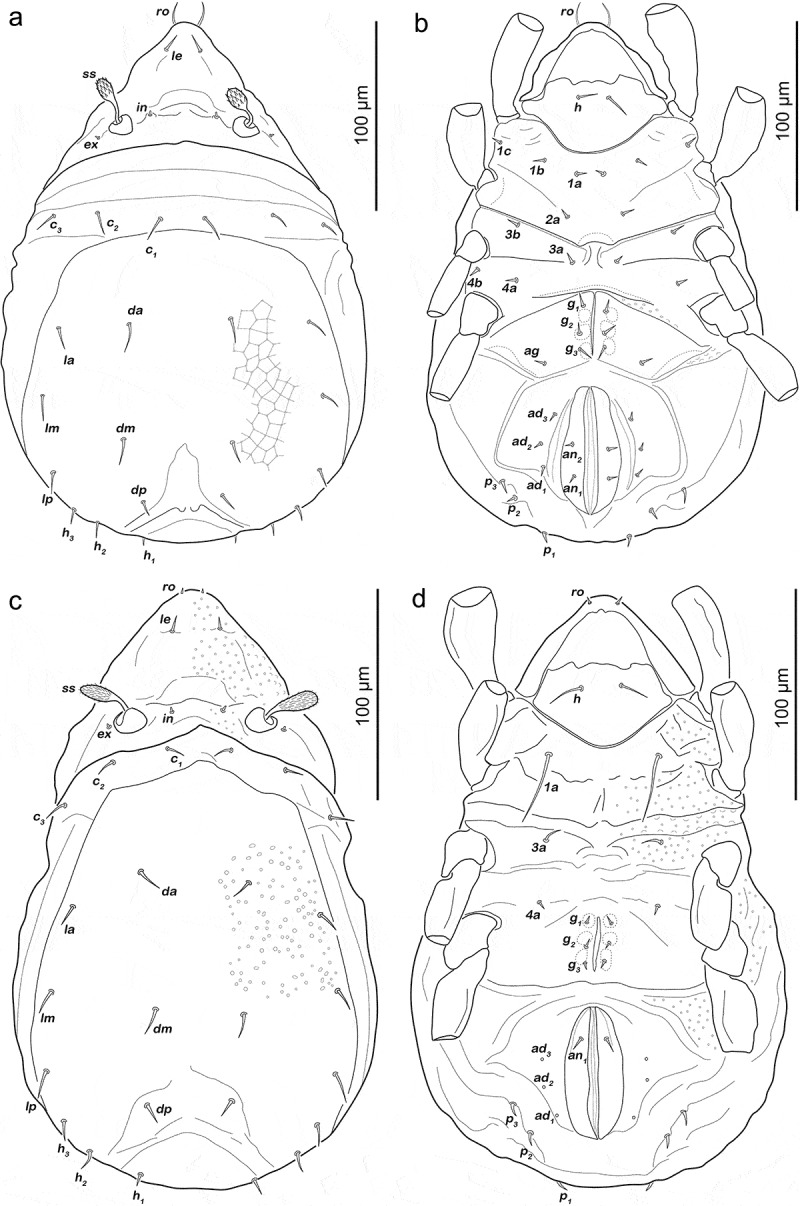
Tritonymphs. (a) *Alismobates reticulatus* dorsal view. (b) *A. reticulatus* ventral view. (c) *Arotrobates granulatus* dorsal view. (d) *A. granulatus* ventral view

Gastronotic region (Figure 10(a)). Fifteen pairs of notogastral setae (8–10 µm), no difference from deutonymph.

Podosoma and venter (Figure 10(b)). Epimeral setation 3-1-2-2, seta *4b* close to trochanter IV. Three pairs of genital setae *g_1-3_*. One pair of aggenital setae. Three pairs of adanal. Two pairs of anal setae, fully developed.

Legs. Setation and solenidia see Table 4.

### Remarks

Adults of *A. reticulatus* are characterized by the name giving reticulate cuticular notogastral pattern, whereas this pattern is best observed around the medial notogastral setae (e.g. Luxton 1992, own observation). The reticulation results from slightly elevated cuticular lines that are connected at various points. Juveniles also show a distinct reticulate pattern on their centrodorsal plate, but the pattern is clearly visible all over this plate (except for the inverted Y on the posterior half) and it results from irregular scale-like elevations (a negative shape from the structure shown in the adults, so to speak). Juveniles of *Alismobates pseudoreticulatus* show the exact same pattern (Pfingstl 2015) but the adults of this species possess only remnants of the reticulate pattern framing the bases of centrodorsal notogastral setae. So, adults can be distinguished based on this trait, while juveniles cannot be. Moreover, we could not find any difference between juveniles of both species that makes an identification on species level impossible.

Family Selenoribatidae Schuster 1963Genus *Arotrobates* Luxton, 1992

### Common features of juvenile stages

Colour brown. Integument plicate and soft, except for centrodorsal plate. Prodorsum triangular, anterior part granular, rostrum rounded. Rostral, lamellar, interlamellar and exobothridial seta*e* short. Sensillum clavate, distally spinose. Bothridium large cup. Large centrodorsal plate bearing centrodorsal setae. Centrodorsal plate slightly convex, plateau-like; lateral and posterior inner borders of plate deepened like a broad furrow. Large folds framing centrodorsal plate completely developed. Ventral sejugal suture developed as distinct furrow but medially interrupted. Other folds typical for selenoribatid juveniles. Legs with large ventral carina on femora, no porous areas detectable. Famulus on tarsus I a short but distinct rod.

***Arotrobates granulatus*** Luxton, 1992

Larva and protonymph unknown.

#### Deutonymph

Length (N = 2): 243–265 (mean 254 μm).

Gastronotic region. Fifteen pairs of normal spiniform notogastral setae (8–12 µm); *c_1-3_, da, dm, dp, la, lm, lp, h_1-3_, p_1-3_*.

Podosoma and venter. Epimeral setation 1-0-1-1. Two pairs of short genital setae (ca. 5 µm) arranged in longitudinal row. Three pairs of vestigial adanal setae flanking anal valves. One pair of vestigial anal setae (*an_1_*).

**Figure 11.**
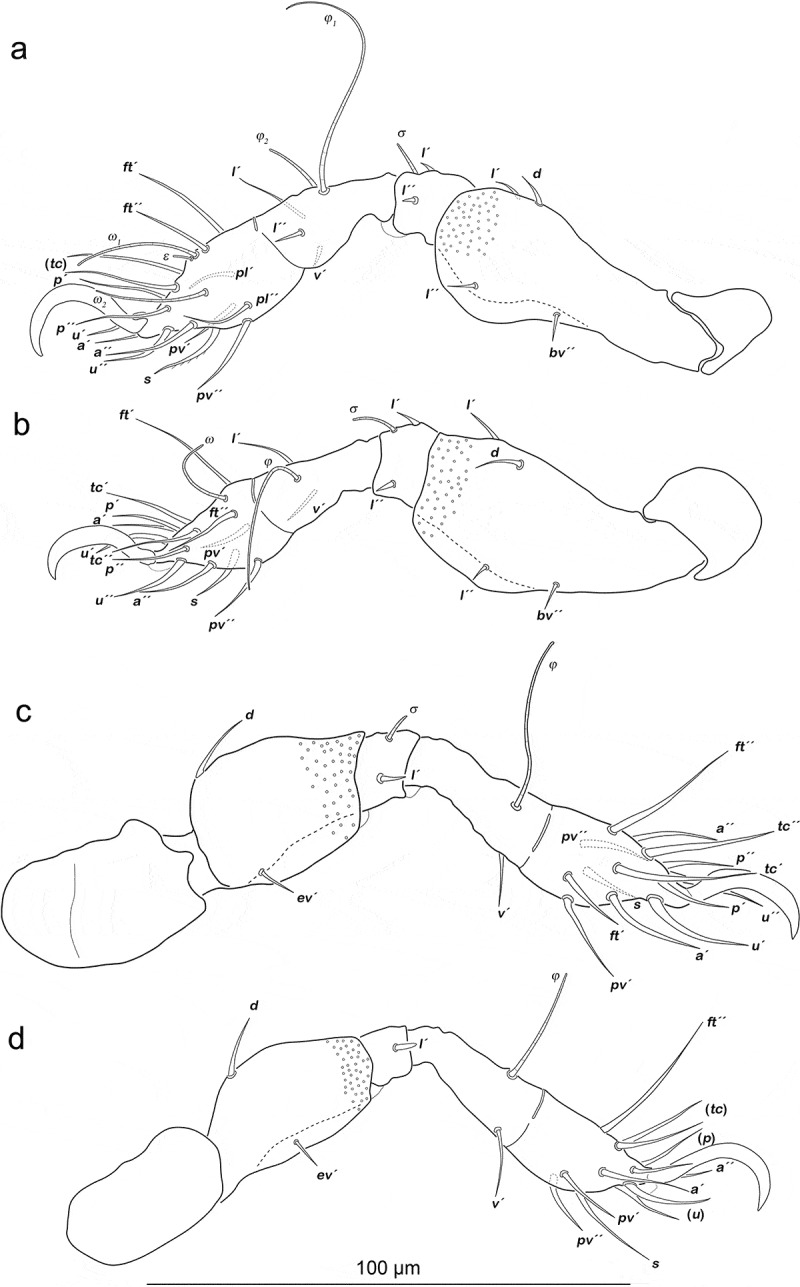
*Arotrobates granulatus* deutonymph, left legs antiaxial view. (a) Leg I. (b) Leg II. (c) Leg III, tibia and tarsus in slight dorsolateral position. (D) Leg IV. Scale bar valid for all legs

Legs (Figure 11). Setation and solenidia are shown in Table 5.

**Table 5.** *Arotrobates granulatus*, chaetome and solenidia from deutonymph to adult

	Instars	Trochanter	Femur	Genu	Tibia	Tarsus	Chaetome	Solenidia
Leg I	deutonymph	-	*d, bv´´*, (*l*)	(*l), σ*	(*l), v´, φ_1_, φ_2_*	(*pl*), (*pv), s*, (*a*), (*u*), (*p*), (*tc*), (*ft), ε, ω_1_, ω_2_*	0-4-2-3-16	1-2-2
	tritonymph	-	-	-	-	(*it*)	0-4-2-3-18	1-2-2
	adult	-	-	-	-	-	0-4-2-3-18	1-2-2
Leg II	deutonymph	-	*d, bv´´*, (*l*)	(*l*), *σ*	*l´, v´, φ*	(*pv), s*, (*a*), (*u*), (*p*), (*tc*), (*ft), ω*	0-4-2-2-13	1-1-1
	tritonymph	-	-	-	-	(*it*)	0-4-2-2-15	1-1-1
	adult	-	-	-	-	-	0-4-2-2-15	1-1-1
Leg III	deutonymph	-	*d, ev´*	*l´, σ*	*v´, φ*	(*pv), s*, (*a*), (*u*), (*p*), (*tc*), (*ft*)	0-2-1-1-13	1-1-0
	tritonymph	-	-	-	-	(*it*)	0-2-1-1-15	1-1-0
	adult	*v´*	-	-	*l´´*	-	1-2-1-2-15	1-1-0
Leg IV	deutonymph	-	*d, ev´*	*l´*	*v´, φ*	(*pv*), (*u*), (*p*), s, (a), (tc), *ft´´*	0-2-1-1-12	0-1-0
	tritonymph	-	-	-	*l´´*	-	0-2-1-2-12	0-1-0
	adult	*v´*	-	-	-	-	1-2-1-2-12	0-1-0

#### Tritonymph

Length (N = 5): 274–299 (mean 285 μm).

Gastronotic region (Figure 10(c)). No difference to deutonymph.

Podosoma and venter (Figure 10(d)). Epimeral setation 1-0-1-1.

Three pairs of short genital setae *g_1-3_* (4–5 µm) in longitudinal row. Three pairs of vestigial adanal setae. Anal setae *an_1_* developed in this stage.

Legs. Setation and solenidia are shown in Table 5.

### Remarks

When Luxton (1992) erected the genus *Arotrobates*, he provided sketchy drawings of the legs of *A. lanceolatus* and mentioned at least in the key that members of this genus show conspicuous crests on all femora. Apart from this info, he provided very few further details on leg features whereas he mostly reported appearances of solenidia, for both known species. Later, Karasawa and Aoki (2005) redescribed *A. granulatus* from the southern and central Ryukyus and also gave only vague information on the legs, e.g. they provided length of each segment and wrote a sentence about pigmentation, but in contrast to Luxton (1992) they did not mention ventral carinae on femora at all. The latter trait, however, is present in the Japanese specimens and represents a prominent feature that is already developed in the juvenile stages. The setation and solenidia, presented herein for the first time in detail, are only based on Japanese *A. granulatus* material. No porous areas could be detected on any leg segment in any stage. Another characteristic trait of *Arotrobates* are the plough-like ventral epimeral carinae and although this feature is not developed in any juvenile stage, the epimeral integument of the nymphs appears to be more folded and less tight than in any other selenoribatid juvenile. This extension of integumental surface could already be an indication of the later developing ventral epimeral carinae.

Genus *Indopacifica* Pfingstl, Shimano & Lienhard, 2019

### Common features of juvenile stages

Colour brown. Integument plicate and soft, except for centrodorsal plate. Whole cerotegument showing dense conspicuous granulation. Prodorsum triangular, rostrum rounded. Rostral, lamellar and interlamellar setae short, exobothridial seta shaped like minute lamella. Sensillum clavate, distally spinose. Bothridium cup-like. Gnathosoma similar to adult stage. Large centrodorsal plate bearing centrodorsal setae. Centrodorsal plate slightly convex, plateau-like; lateral and posterior inner borders of plate deepened like broad furrow. Large folds framing centrodorsal plate completely. Ventral furrows typical for selenoribatid juveniles. Legs, tarsus I with short knob-like famulus, no porose areas on femora detectable.

***Indopacifica taiyo*** Pfingstl, Shimano & Hiruta, 2021

Larva unknown.

#### Protonymph

Length (N = 2): 194 μm (both the same size).

Gastronotic region. Fifteen pairs of notogastral setae; *c_1-3_, da, dm, dp, la, lm, lp, h_1-3_* and *p_1-3_*, all short, approximately same length (8–10 µm).

Podosoma and venter. Epimeral setation 1-0-1-1. Seta *4a* medially on epimeron IV. One pair of short genital setae. Aggenital setae absent.

Legs. Claws with one proximoventral tooth. Setation and solenidia see Table 6.

**Table 6.** *Indopacifica taiyo*, chaetome and solenidia from protonymph to adult (tritonymph excluded due to missing data). *information for adult taken from Pfingstl et al. (2021)

	Instars	Trochanter	Femur	Genu	Tibia	Tarsus	Chaetome	Solenidia
Leg I	protonymph	-	*d, bv´´, l*´	(*l), σ*	*l´, v´, φ_1_*	(*pl*), (*pv), s*, (*a*), (*u*), (*p*), (*tc*), (*ft), ε, ω_1_, ω_2_*	0-3-2-2-16	1-1-2
	deutonymph	-	-	-	*φ_2_*	-	0-3-2-2-16	1-2-2
	adult*	-	-	-	*l´´*	(*it*)	0-3-2-3-18	1-2-2
Leg II	protonymph	-	*d, bv´´, l*´	(*l*), *σ*	*l´, v´, φ*	(*pv*), *s*, (*a*), (*u*), (*p*), (*tc*), (*ft), ω*	0-3-2-2-13	1-1-1
	deutonymph	-	-	-	-	-	0-3-2-2-13	1-1-1
	adult*	-	-	-	*l´´*	(*it*)	0-3-2-3-15	1-1-1
Leg III	protonymph	-	*d, ev´*	*l´, σ*	*v´, φ*	(*pv), s*, (*a*), (*u*), (*p*), (*tc*), (*ft*)	0-2-1-1-13	1-1-0
	deutonymph	-	-	-	-	-	0-2-1-1-13	1-1-0
	adult*	*v´*	-	-	*l´*	(*it*)	1-2-1-2-15	1-1-0
Leg IV	protonymph	-	-	-	-	(*pv*), (*u*), (*p), ft´´*	0-0-0-0-7	0-0-0
	deutonymph	-	*d, ev´*	*l´*	*v´, φ*	*s*, (*a*), (*tc*)	0-2-1-1-12	0-1-0
	adult*	*v´*	-	-	(*l*)	-	1-2-1-3-12	0-1-0

#### Deutonymph

Length (N = 5): 206–237 (mean 227 μm).

**Figure 12.**
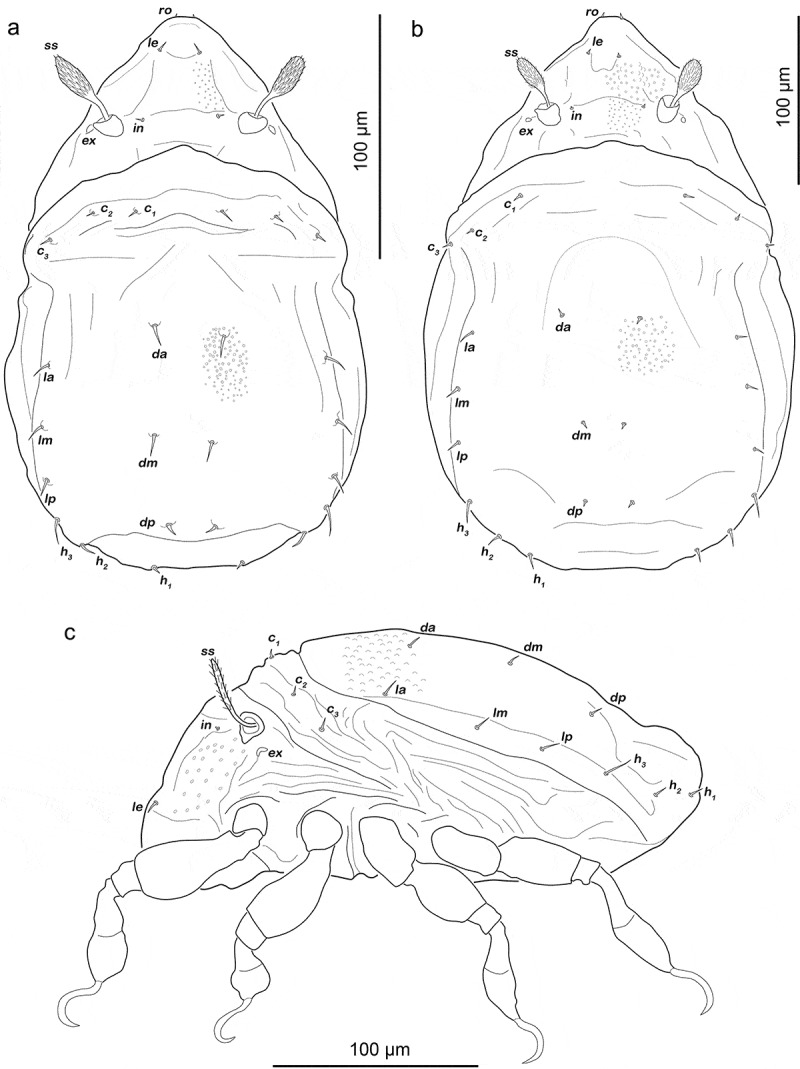
Japanese *Indopacifica* nymphs. (a) *I. taiyo* deutonymph dorsal view, legs omitted. (b) *I. tyida* tritonymph dorsal view, legs omitted. (c) *I. tyida* deutonymph lateral view, legs drawn simplified

Gastronotic region (Figure 12(a)). Fifteen pairs of notogastral setae, same positions and shapes as in protonymph.

Podosoma and venter. Epimeral setation 1-0-1-1. Two pairs of short genital setae (approx. 4 µm) arranged in a longitudinal row. Two pairs of adanal setae *ad_1-2_* flanking anal valves. Two pairs of vestigial anal setae.

Legs. Claws with one proximoventral tooth. Setation and solenidia are shown in Table 6.

Tritonymph unknown.

***Indopacifica tyida*** Pfingstl, Shimano & Hiruta, 2021

Larva and protonymph unknown.

#### Deutonymph

Length (N = 3): 250–295 (mean 276 μm).

Gastronotic region (Figure 12(c)). Fifteen pairs of notogastral setae; *c_1-3_, da, dm, dp, la, lm, lp, h_1-3_* and *p_1-3_*, all short, approximately same length (6–10 µm).

Podosoma and venter. Epimeral setation 1-0-1-1. Two pairs of short genital setae (ca. 4 µm) arranged in longitudinal row. Two pairs of short adanal setae *ad_1-2_* (ca. 3 µm) flanking anal valves. Two pairs of vestigial anal setae.

**Figure 13.**
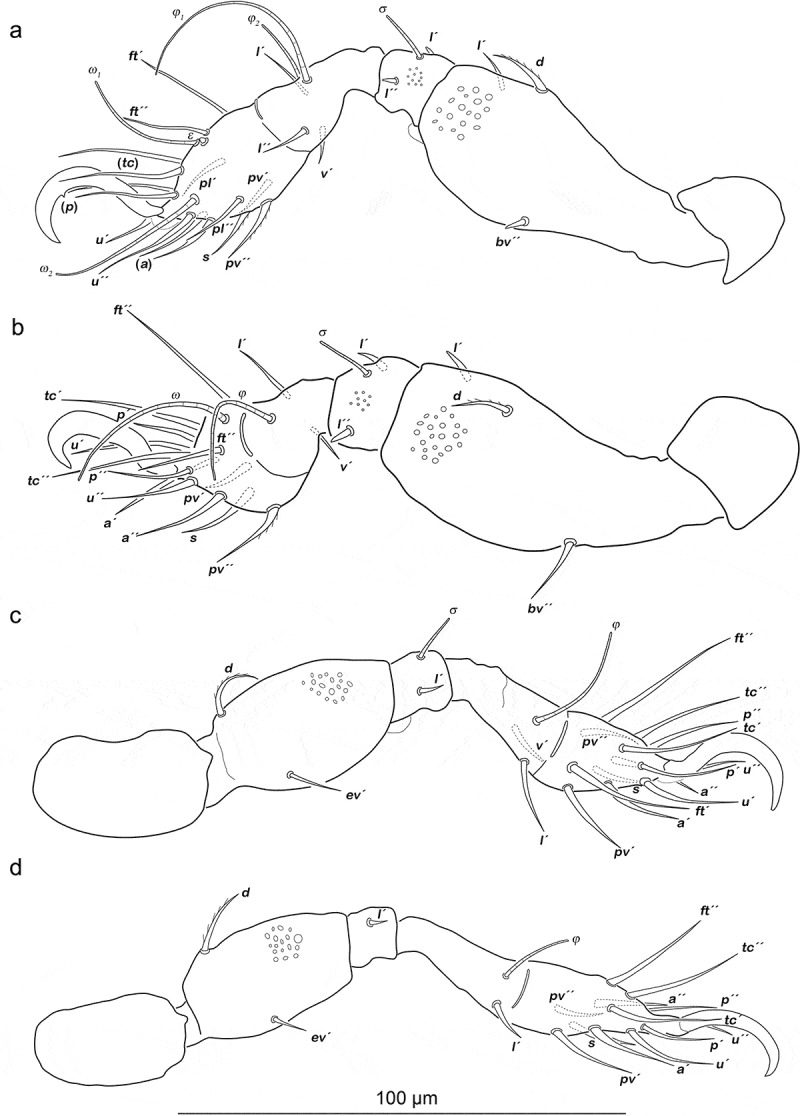
*Indopacifica tyida* deutonymph, left legs antiaxial view (legs II–IV slightly dorsolaterally twisted). (a) Leg I. (b) Leg II. (c) Leg III. (d) Leg IV. Scale bar valid for all legs. Proximoventral tooth on claws only visible on leg I, due to unfavourable inclination angle of view it is not shown on other legs

Legs (Figure 13). Claws with one proximoventral tooth. Setation and solenidia are shown in Table 7.

**Table 7.** *Indopacifica tyida*, chaetome and solenidia from deutonymph to adult. *information for adult taken from Pfingstl et al. (2021)

	Instars	Trochanter	Femur	Genu	Tibia	Tarsus	Chaetome	Solenidia
Leg I	deutonymph	-	*d, bv´´, l*´	(*l), σ*	(*l), v´, φ_1_, φ_2_*	(*pl*), (*pv), s*, (*a*), (*u*), (*p*), (*tc*), (*ft), ε, ω_1_, ω_2_*	0-3-2-3-16	1-2-2
	tritonymph	-	-	-	-	(*it*)	0-3-2-3-18	1-2-2
	adult*	-	-	-	-	-	0-3-2-3-18	1-2-2
Leg II	deutonymph	-	*d, bv´´, l*´	(*l*), *σ*	*l´, v´,φ*	(*pv), s*, (*a*), (*u*), (*p*), (*tc*), (*ft), ω*	0-3-2-2-13	1-1-1
	tritonymph	-	-	-	-	(*it*)	0-3-2-2-15	1-1-1
	adult*	-	-	-	*l´´*	-	0-3-2-3-15	1-1-1
Leg III	deutonymph	-	*d, ev´*	*l´, σ*	*v´, l´, φ*	(*pv), s*, (*a*), (*u*), (*p*), (*tc*), (*ft*)	0-2-1-2-13	1-1-0
	tritonymph	-	-	-	-	(*it*)	0-2-1-2-15	1-1-0
	adult*	*v´*	-	-	-	-	1-2-1-2-15	1-1-0
Leg IV	deutonymph	-	*d, ev´*	*l´*	*l´, φ*	(*pv*), (*u*), (*p*), s, (a), (tc), *ft´´*	0-2-1-1-12	0-1-0
	tritonymph	-	-	-	*v´*	-	0-2-1-2-12	0-1-0
	adult*	*v´*	-	-	*l´´*	-	1-2-1-3-12	0-1-0

#### Tritonymph

Length (N = 1): 325 μm.

Gastronotic region (Figure 12(b)). No difference to deutonymph.

Podosoma and venter. Epimeral setation 1-0-1-1. Three pairs of short genital setae (4 µm) in longitudinal row. Two pairs of adanal setae and two pairs of short anal setae *an_1-2_* (4 µm).

Legs. Claws with one proximoventral tooth. Setation and solenidia are shown in Table 7.

### Remarks

Juveniles of the Japanese *Indopacifica* species are more or less identical in terms of habitus, prodorsal, notogastral and ventral setation. The only found differences between *I. taiyo* and *I. tyida* juveniles concern the development of tibial setae, i.e. the antiaxial lateral setae on tibia I and III appear already in the deutonymphal stage in *I. tyida* but they appear later (possibly in tritonymph but due to lacking data not confirmed yet) in *I. taiyo*.

## Discussion

Grandjean (1954) organized the Brachypylina using the conservatism of juvenile morphology. Indeed, juveniles show more conservative morphologies that may allow to infer high-level relationships (e.g. Norton and Ermilov 2014). On the other hand, this conservatism may mask relationships between species so that it may be impossible to differentiate two closely related species based on their juveniles (e.g. Pfingstl and Krisper 2011) and therefore juvenile morphology is less suitable for species determination. Ermilov et al. (2012) provided diagnostic keys for ameronothrid juveniles but only to generic level for the reason given above. Herein, we even refrain from giving any diagnostic key because of strongly overlapping characteristics in selenoribatid juvenile instars and because of missing data from several genera, e.g. *Psednobates, Rhizophobates, Thasecazetes*.

Nevertheless, certain taxonomic and phylogenetic statements can be drawn from present data. First of all, juveniles of *Alismobates reticulatus* and *A. pseudoreticulatus* show a distinct reticulate pattern on their centrodorsal plate and this pattern is also present in the adults. All other known *Alismobates* juveniles show a clear foveate pattern that is absent in the adult stage. Consequently, *A. reticulatus* and *A. pseudoreticulatus* may share a common ancestor and the reticulate pattern may represent a synapomorphy of these two species. *Alismobates reticulatus* is distributed from Hong Kong (Luxton 1992) to the southern Japanese islands and *A. pseudoreticulatus* was reported from Thailand and the Malayan peninsula (Pfingstl 2015; Pfingstl et al. 2019a) and together with *A. rotundus* they are the only species of this genus reported from Southeast and East Asia. The latter species was reported from Hong Kong but unfortunately juveniles are unknown and thus it remains unclear if they also show a reticulate centrodorsal plate and if this trait relates to a phylogeographic lineage.

Adults of the genus *Fortuynia* show a consistently homogeneous morphology with very few distinguishing characters (Pfingstl 2015) and this homogeneity is even more pronounced in the immature stages. In 10 out of 16 described species, immatures share the same habitus, colouration and developmental formulas of notogastral, epimeral and anogenital setae. Variation between juveniles of different species can mainly be found in the length of setae and the presence or absence of barbs on notogastral setae. An unusual exception are nymphs of *Fortuynia arabica* Bayartogtokh, Chatterjee, Chan & Ingole, 2009 and *F. taiwanica* Bayartogtokh, Chatterjee, Chan & Ingole, 2009, because both species show obvious deviations in certain developmental patterns (Bayartogtokh et al. 2009). The deutonymph of *F. arabica* exhibits only 10 gastronotic setae whereas the tritonymph shows the full set with 15 setae (Bayartoghtokh et al. 2009), all other *Fortuynia* juveniles already show the full set of gastronotic setae from the protonymphal stage and never show any reduction during development. The authors based their observation on a single deutonymphal specimen that is absolutely okay if there is only one specimen available but it could be that this specimen represents an aberrant individual. This individual shows another striking deviation as it possesses three genital setae but lacks the aggenital seta (Bayartogtokh et al. 2009), whereas all other *Fortuynia* deutonymphs show only two genital setae and the presence of one aggenital seta. We think that this deviation is based on a simple mistake because the third genital seta of the *F. arabica* deutonymph could be the aggenital seta that usually appears in the deutonymph. The protonymph of *F. taiwanica* shows another developmental abnormality because it already shows one pair of adanal setae (Bayartogtokh et al. 2009). These setae usually appear not before the deutonymphal stage. However, this adanal seta is absent in the ventral depiction of the protonymph of *F. taiwanica* (Bayartogtokh et al. 2009, p. 249, Figure 4(b)) so it is possible that the authors just made a little slip in the text causing this unusual divergence.

The present description of the juvenile stages of *Arotrobates granulatus* represents the first report of immature stages in the genus and thus we cannot compare it to larvae and nymphs of congeneric species. However, nymphal characteristics of *A. granulatus* are in accordance with juvenile traits of Selenoribatidae (Grandjean 1966) confirming its systematic position and nymphs can hardly be distinguished from any other selenoribatid juvenile at first glance. Possible diagnostic characters for juveniles of *Arotrobates* are only the more folded and less tight anterior epimeral integument and the very prominent ventral femoral carinae. The former is supposed to be an indication of the later developing adult ventral epimeral carinae. The genus *Carinozetes* shows similar epimeral carinae in the adult stage but nymphs lack any similarity with *Arotrobates* nymphs in this respect. The other trait, femoral ventral carinae are a widespread feature among Selenoribatidae and certain species, e.g. *Schusteria littorea, Thalassozetes canariensis*, are known to show this feature early in their development (Grandjean 1968; Pfingstl et al. 2020) but adults as well as nymphs of *Arotrobates granulatus* possess more prominent carinae than any other known selenoribatid taxon. Nonetheless, these characters are difficult to assess without direct comparison and it is unclear if they are also present in *Arotobates lanceolatus* juveniles; therefore, they should be viewed with caution.

Similar to *Fortuynia*, adults of the genus *Indopacifica* show a remarkable homogeneous morphology with very few characters allowing determination (Pfingstl et al. 2021) and the same applies to the juveniles. Immature stages of *I. iohanna, I. taiyo* and *I. tyida* show conformity in all characters except for the legs. *Indodpacifica iohanna* juveniles can be distinguished by the lack of proximoventral teeth on the claws (Resch et al. 2019), a character that is present in the juveniles of the two other species. *Indopacifica taiyo* and *I. tyida* show slight differences in the development of leg setation (details are outlined in the respective remarks section), which would allow distinction at least in the deutonymphal stage. Basically, all *Indopacifica* juveniles show a strongly granular cerotegument, a feature that is only similarly shown in *A. granulatus* nymphs, and they all possess exobothridial setae that are shaped like a minute lamella. The latter trait may be diagnostic for the juveniles of this genus but such tiny structures are often misinterpreted in light microscopic view and thus need further confirmation with other means of observation.
